# Meta-analysis of trimetazidine treatment for cardiomyopathy

**DOI:** 10.1042/BSR20171583

**Published:** 2018-06-12

**Authors:** Qian Fan, Zhaozhuo Niu, Liqing Ma

**Affiliations:** 1 Department of Cardiovasology, Qingdao Municipal Hospital, School of Medicine, Qingdao University, Qingdao 266011, China; 2 Department of Cardiac Surgery, Qingdao Municipal Hospital, School of Medicine, Qingdao University, Qingdao 266011,China; 3 Department of Geriatrics, Qingdao Municipal Hospital, School of Medicine, Qingdao University, Qingdao 266011, China

**Keywords:** Cardiomyopathy, Meta-Analysis, publication bias, Trimetazidine

## Abstract

To explore the effect of trimetazidine (TMZ) in cardiomyopathy treatment. Literatures, related with TMZ treatment for cardiomyopathy, were retrieved between 1990 and February 2018 in the Pubmed, Embase, and Cochrane Library systems. Cardiopulmonary exercise testing [resting heart rate (RHR), peak heart rate (PHR), peak systolic blood pressure (PSBP), and resting systolic blood pressure (RSBP)] and echocardiographic results [left ventricular ejection fraction (LVEF), left ventricular end-systolic volume (LVESV), left ventricular end-diastolic volume (LVEDV), systolic wall thickening score index (SWTSI), left ventricular end-systolic diameter (LVESD), and left ventricular end-diastolic diameter (LVEDD)] were merged to detect the publication bias. Total 898 patients with cardiomyopathy were divided into two groups: TMZ-treated group (*n*=456) and control group (*n*=442). There was no difference in the improvement of cardiomyopathy between the TMZ and control group. No publication bias was shown for PHR (*t*= 0.9791, *P*=0.5067). There were significant differences in LVEF, LVESV, SWTSI, LVESD, and LVEDD between the TMZ group and the control group. TMZ-treatment significantly increased the level of LVEF (95% confidence interval (CI): 5.46–7.84, *P*<0.001), and reduced the level of LVESV (95% CI: −18.73 to −7.77, *P*<0.001), SWTSI (95% CI: −0.47 to −0.15, *Z* = −3.85, *P*=0.001), LVESD (95% CI: −1.09 to −0.08, *P*<0.001), and LVEDD (95% CI: −0.55 to −0.26, *P*=0.023). There was no publication bias except for LVEDV (*t* = 2.5456, *P*=0.0438). TMZ is effective for cardiomyopathy treatment and worth to popularize in clinic.

## Introduction

Cardiomyopathy, defined as myocardial disease associated with cardiac functional insufficiency, is divided into primary and secondary cardiomyopathies [1]. The manifestations include exertional dyspnea, chest pain, arrhythmia, syncope, and even sudden death [2]. Etiology of primary cardiomyopathy is unknown so far. It is generally believed that cardiomyopathy is a clinical syndrome with multiple etiologies, such as viral infection, immune response, genetic theory, myocardial ischemia, metabolism and enzyme changes, catecholamine theory, poisoning theory, and nutritional deficiency [3]. Trimetazidine (TMZ), a piperazine derivative which acts on myocardium metabolism, inhibits mitochondrial long-chain acyl coenzyme 3-ketone A thiolase, transfers the energy from metabolism of fatty acid oxidation to glucose oxidation, uses limited oxygen to produce more ATP, and increases the synthesis of phospholipids, so it makes the myocardial cell energy production optimization. [4]. TMZ could stimulate glucose metabolism to increase the myocardial tolerance to ischemic injury by inhibiting the β-oxidation pathway of fatty acids [5].

At present, trimetazidine was widely used in the treatment of cardiomyopathy, but there were no consistent reports on the efficacy of TMZ for cardiomyopathy treatment. Therefore, it is necessary to make a comprehensive evaluation of the efficacy of TMZ in the treatment of cardiomyopathy by means of meta-analysis, providing a basis for the clinical treatment of cardiomyopathy.

## Methods

### Data sources

The comprehensive search was performed to retrieve the related clinical studies in Pubmed (http://www.ncbi.nlm.nih.gov/pubmed), Embase (http://www.embase.com), and Cochrane Library (http://www.cochranelibrary.com) with the following key words: ‘cardiomyopathies’ or ‘cardiomyopathy’ or ‘myocardiopathy’ in combination with ‘TMZ’ or ‘trimetazine’ or ‘vastarel’ or ‘vasorel’. The study type is a randomized controlled study, the study language is limited to english, and the last search was updated on February 2018.

### Inclusion criteria

Inclusion criteria for studies were shown as follows: (1) published literature on the clinical study of TMZ therapy in cardiomyopathy; (2) the study type is a randomized controlled study; (3) it could provide the comparison of the efficacy between TMZ and control groups in the treatment of cardiomyopathy; (4) mainly included some outcome indicators of cardiopulmonary exercise testing and echocardiographic.

Exclusion criteria were as follows: (1) data were not complete or not available for statistical analysis; (2) non-original studies, such as reviews, letters etc.; (3) duplicate publication; (4) the data from the same population used for multiple studies are only included in the latest research or one of the most complete information, with the remainder excluded.

### Data extraction and quality assessment

Two authors (F.Q. and N.Z.Z.) independently extracted the following information of studies: first authors, publication year, research area, follow-up time, the patients number of the TMZ and control group, age and gender of patients, and left ventricular ejection fraction (LVEF) indexes before treatment and outcome related (cardiopulmonary exercise testing and echocardiographic).

Quality assessment was processed using the evaluation instrument recommendated by Cochrane Collaboration for bias risk assessment.

If there are differences of opinion, discrepancy between the two investigators, the third investigator was invited to discuss for consensus.

### Statistical analysis

All procedures of meta-analysis were performed using R 3.12 software (R Foundation for Statistical Computing, Beijing 1, China, meta package). The quantitative data effect index is represented by mean difference (MD) and its 95% confidence interval (CI). The heterogeneity test, which used to check whether the results of individual studies are unifiable, was analyzed by *Q* test based on Chi square [6] and *I^2^* statistic. If the heterogeneity test was statistically significant (*P*<0.05 or *I^2^* > 50%), the random effect model (REM) was used to calculate the combined effect value; otherwise, fixed effect model (FEM) was used to merge data (*P*<0.05 and *I^2^* > 50%) [7]. Egger’s method was used to test publication bias.

## Result

### General features of selected literature

**Figure 1:**
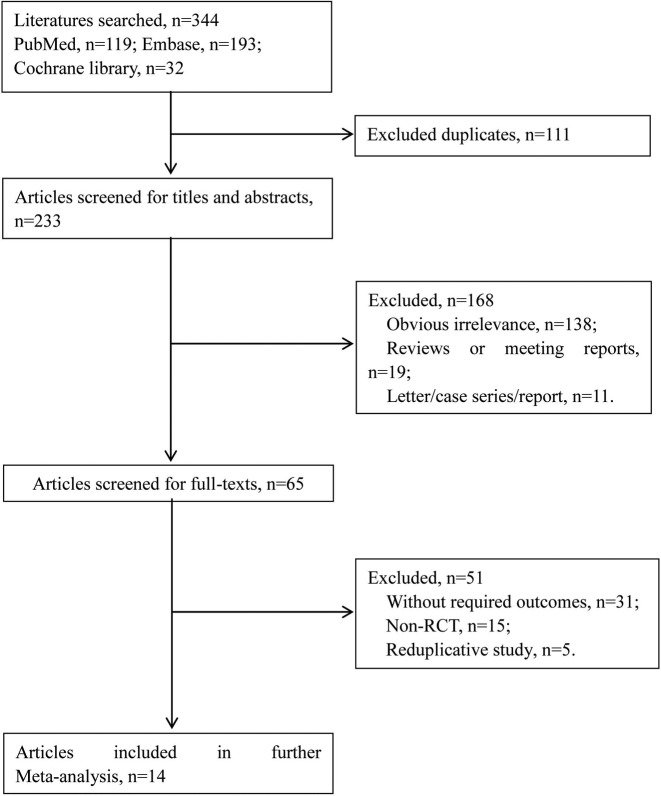
The search results and selection process of literature

The search results and selection process of literature were shown in Figure 1. Total 344 articles were retrieved by using the retrieval strategy in Pubmed, Embase, and Cochrane Library databases. After eliminating the repetitive literatures (111), 233 articles were remained. After browsing the headlines and abstracts, 168 papers were excluded, including 138 articles which did not meet the inclusion criteria, 19 reviews or conference papers, and 11 letters, case series or report. Among the 65 remaining articles, 51 articles were excluded after reading the full text, and total 14 qualified articles were included [8–21].

**Figure 2:**
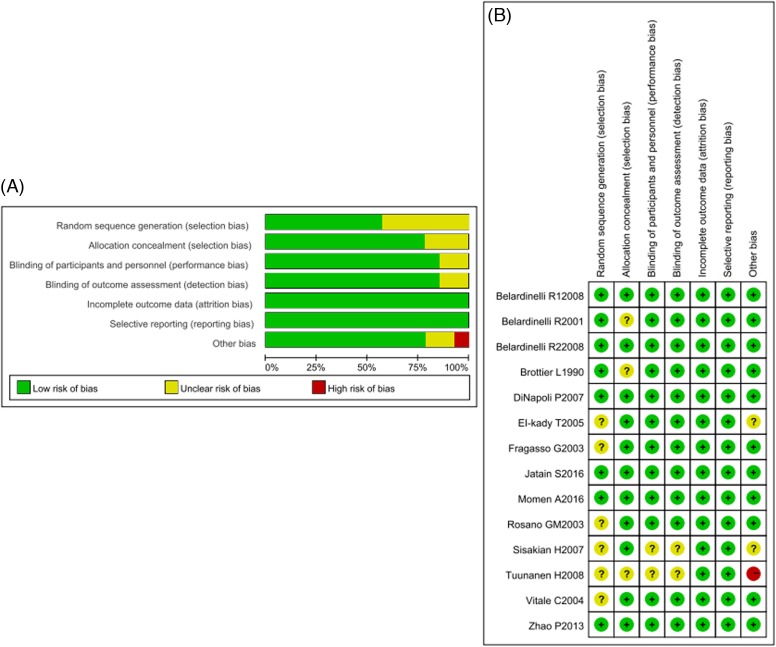
Risk of bias graph and risk of bias summary (**A**) Risk of bias graph: the judgment of the percentage of all projects that have the risk of bias in the study. (**B**) Risk of bias summary: judgment of all the risk items of each bias in the study. Notes: Green: low risk of bias; Yellow: unclear risk of bias; Red: high risk of bias.

The general features of the selected literature were shown in Table 1. Total 898 patients with cardiomyopathy were included, including TMZ group (*n*=456) and control group (*n*=442). Selected articles published between 1990 and 2016, and most published after 2001 (13/14). The study area is mainly in France, Italy, Denmark, Egypt, and China, the follow-up time was usually 3–6 months, the patients in included studies were mainly male with middle-aged or elderly. General conditions of these patients, including gender, age, and LVEF before treatment were not statistically different between two groups. The relevant outcome measures (cardiopulmonary exercise testing and echocardiographic) were recorded in Table 2. The quality evaluation was shown in Figure 2. It could be seen that the overall quality of articles was higher.

**Table 1:** The general features of the selected literature

Author	Publication year	Study location	Follow-up	Group	*N*	Male	Age	LVEF(%)
Belardinelli, R.[8]	2008	Italy	3 months	T	19	16	54.3 ± 9	39 ± 6
				C	15	14	53.7 ± 9	40 ± 6
Belardinelli, R.[9]	2008	Italy	8 weeks	T	30	25	59 ± 8	35 ± 7
				C	26	22	58 ± 9	36 ± 6
Belardinelli, R.[10]	2001	Italy	6 months	T	19	15	50 ± 7	33 ± 4.5
				C	19	16	54 ± 7	33.4 ± 3.5
Brottier, L.[11]	1990	France	6 months	T	9	NA	57.0 ± 3.2	32.2 ± 3.1
				C	11	NA	61.9 ± 0.9	29.4 ± 2.6
DiNapoli, P.[12]	2007	Italy	6 months	T	25	15	64 ± 6	28 ± 4
				C	25	18	63 ± 7	30 ± 6
EI-kady, T.[13]	2005	Egypt	24 months	T	100	86	52.8 ± 8.7	35.6 ± 17.1
				C	100	78	53.1 ± 8.7	36.9 ± 13.9
Fragasso, G.[14]	2003	Canada	6 months	T	16	16	64 ± 7	NA
				C	16	16		
Jatain, S.[16]	2016	India	6 months	T	50	73	47.1 ± 12.6	27.0 ± 6.2
				C	50	75	48.31 ± 11.5	27.6 ± 4..2
Momen, A.[17]	2016	Bangladesh	6 months	T	55	45	58 ± 9.5	32.9 ± 6.6
				C	53	41	59 ± 8.9	33.1 ± 6.2
Rosano, G.M.[15]	2003	Italy	6 months	T	16	11	65.6 ± 5.7	32.3 ± 5.3
				C	16	13	65.2 ± 7	32.8 ± 2.3
Sisakian, H.[18]	2007	Armenia	3 months	T	42	37	63.5 ± 9.3	34.5 ± 3.8
				C	40	33	65.6 ± 8.7	32.4 ± 5.6
Tuunanen, H.[19]	2008	France	3 months	T	12	10	59 ± 8.8	31 ± 8.5
				C	7	5	57 ± 7.3	38 ± 8.4
Vitale, C.[20]	2004	Italy	6 months	T	23	18	77 ± 2.3	29 ± 2.2
				C	24	22	78 ± 4.3	28.7 ± 2.8
Zhao, P.[21]	2013	China	6 months	T	40	32	59 ± 8.1	34 ± 8.5
				C	40	30	58 ± 9.0	36 ± 8.4

Abbreviations: C, control group; T, trimetazidine group.

**Table 2:** Outcome data

Author	Publication year	Group	*N*	RHR	PHR	RSBP	PSBP	LVEF	LVESV	LVEDV	SWTSI	LVESD	LVEDD
Belardinelli, R.[8]	2008	T	19	NA	NA	NA	NA	43 ± 6	NA	NA	1.7 ± 0.9	NA	NA
		C	15	NA	NA	NA	NA	38 ± 6	NA	NA	2.3 ± 0.9	NA	NA
Belardinelli, R. [9]	2008	T	30	75.0 ± 10.0	137.0 ± 18.0	NA	157.0 ± 19.0	40.5 ± 7.0	88.0 ± 11.0	152.0 ± 18.0	1.70 ± 0.5	4.6 ± 0.6	6.31 ± 0.8
		C	26	79.0 ± 11.0	134.0 ± 18.0	NA	145.0 ± 19.0	36.0 ± 6.0	95.0 ± 9.0	148.0 ± 17.0	1.92 ± 0.5	4.9 ± 0.7	6.44 ± 0.7
Belardinelli, R. [10]	2001	T	19	79 ± 9	87 ± 8	108 ± 21	135 ± 20	42·9 ± 4·4	97·7 ± 11·6	171·7 ± 13	1·41 ± 0·09	NA	NA
		C	19	81 ± 10	89 ± 7	110 ± 22	132 ± 10	38·9 ± 3·5	108 ± 11·8	170·5 ± 19	1·83 ± 0·16	NA	NA
Brottier, L.[11]	1990	T	9	NA	NA	NA	NA	29.6 ± 3.2	NA	NA	NA	NA	NA
		C	11	NA	NA	NA	NA	18.6 ± 2.0	NA	NA	NA	NA	NA
DiNapoli, P.[12]	2007	T	25	NA	NA	NA	NA	32 ± 5	NA	NA	NA	NA	NA
		C	25	NA	NA	NA	NA	26 ± 7	NA	NA	NA	NA	NA
EI-kady, T.[13]	2005	T	100	NA	NA		NA	43.9 ± 21.2	NA	NA	NA	NA	NA
		C	100	NA	NA		NA	37.1 ± 14.0	NA	NA	NA	NA	NA
Fragasso, G.[14]	2003	T	16	NA	NA	NA	NA	44.8 ± 7.5	85.0 ± 36.5	150.3 ± 47.9	NA	5.27 ± 0.96	6.52 ± 0.70
		C	16	NA	NA	NA	NA	36.4 ± 8.0	98.8± 47.5	152.9 ± 57.9	NA	5.62 ± 0.92	6.99 ± 0.88
Jatain, S.[16]	2016	T	50	NA	NA	NA	NA	34.84 ± 8.10	NA	NA	NA	4.51 ± 0.71	6.00 ± 0.65
		C	50	NA	NA	NA	NA	27.69 ± 5.56	NA	NA	NA	5.03 ± 0.62	6.36 ± 0.66
Momen, A.[17]	2016	T	55	NA	NA	NA	NA	36.60 ± 5.5	NA	NA	NA	NA	5.97± 0.52
		C	53	NA	NA	NA	NA	31.20 ± 6.4	NA	NA	NA	NA	6.51 ± 0.61
Rasano, G. M.[15]	2003	T	16	NA	NA	NA	NA	37.7 ± 5.7	NA	NA	NA	3.41 ± 0.09	5.72 ± 0.21
		C	16	NA	NA	NA	NA	30.4 ± 2.9	NA	NA	NA	4.11 ± 0.27	6.32 ± 0.47
Sisakian, H.[18]	2007	T	42	NA	NA	NA	NA	38.0 ± 4.8	120 ± 18	194 ± 25	NA	NA	NA
		C	40	NA	NA	NA	NA	33.2 ± 5.8	139 ± 21	208 ± 27	NA	NA	NA
Tuunanen, H.[19]	2008	T	12	60 ± 9	NA	119 ± 12	NA	34.8 ± 12	204 ± 131	296 ± 129	NA	6.1 ± 0.10	7.4 ± 0.90
		C	7	61 ± 13	NA	116 ± 10	NA	31.9 ± 12	186 ± 76	268 ± 89	NA	5.8 ± 0.62	7.0 ± 0.64
Vitale, C.[20]	2004	T	23	NA	NA	NA	NA	34.4 ± 2.3	73.4 ± 4.2	111.9 ± 3.1	1.24 ± 0.12	4.45 ± 0.11	5.86 ± 0.19
		C	24	NA	NA	NA	NA	27 ± 2.8	90.8 ± 7.2	124.3 ± 6.8	1.45 ± 0.19	5.0 ± 0.08	6.4 ± 0.17
Zhao, P.[21]	2013	T	40	NA	NA	NA	NA	46 ± 9.8	NA	NA	NA	3.1 ± 0.2	5.91 ± 0.7
		C	40	NA	NA	NA	NA	36 ± 7.5	NA	NA	NA	4.9 ± 0.3	6.11 ± 0.6

Abbreviations: LVESDD, left ventricular end-diastolic diameter; LVEDV, left ventricular end-diastolic volume; LVESD, left ventricular end-systolic diameter; LVESV, left ventricular end-systolic volume; PHR, peak heart rate; PSBP, peak systolic blood pressure; RHR, resting heart rate; RSBP, resting systolic blood pressure; SWTSI, systolic wall thickening score index.

### Quantitative data consolidation

First of all, the heterogeneity test was carried out. Then the proper effect model was used to calculate the combined effect value according to the *P*-value of the *Q* test and the *I^2^* statistic.

### Cardiopulmonary exercise testing results

**Figure 3:**
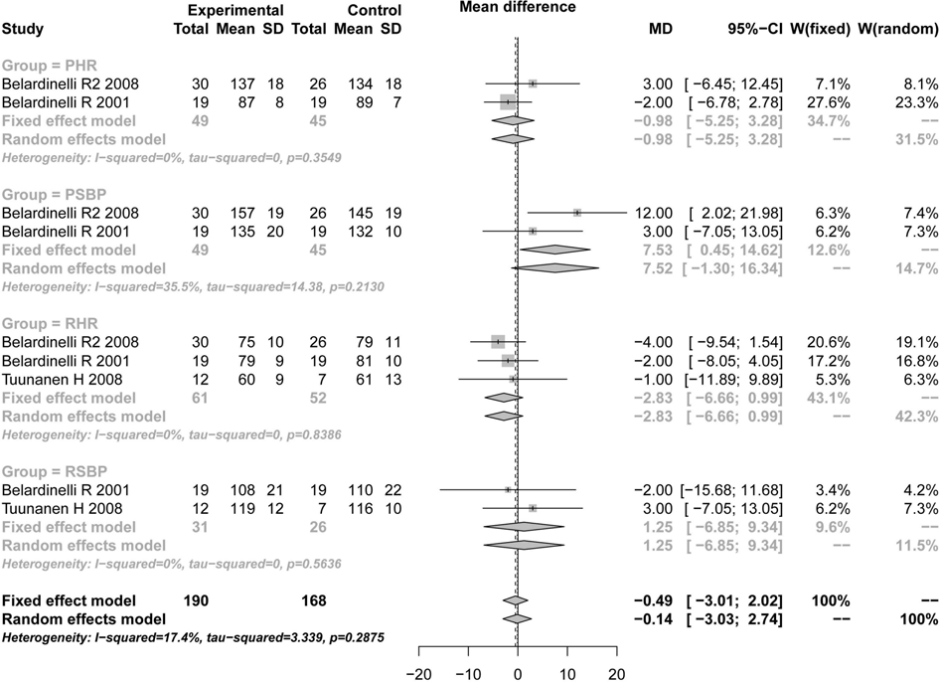
Forest plot for PHR, PSBP, RHR, and RSBP of TMZ group and control group

Indexes including PHR, PSBP, RHR, and RSBP were conformed to the condition of fixed utility model (*P*<0.05 and *I^2^* > 50%), fixed utility models were used for merging. The results showed that PHR (TMZ group 49, control group 45, MD = −0.98, 95% CI: −5.25 to 3.29, *Z* = 1.45, *P*= 0.1470), PSBP (TMZ group 49, control group 45, MD = 7.53, 95% CI: 0.45–14.62, *Z* = −0.45, *P*=0.6519), RHR (TMZ group 61, control group 52, MD = −2.83, 95% CI: −6.66 to 0.99, *Z* = 0.30, *P*=0.7628), and RSBP (TMZ group 31, control group 26, MD = 1.25, 95% CI: −6.85 to 9.34, *Z* = 1.67, *P*=0.0946). There was no difference in the improvement of cardiomyopathy between the TMZ and control group. After assessing by the ‘Egger’s’ method, no publication bias was shown for PHR (*t* = 0.9791, *P*=0.5067) (Table 3 and Figure 3).

**Table 3:** The results of meta-analysis

Variables	Group	Sample size			Test of association			Model	Test of heterogeneity^*,†^			Egger’s test^‡^	
		*K*	Cases	Control	MD [95% CI]	*Z*	*P* -value		*Q*	*P* -value	*I^2^*(%)	*t*	*P-* value
Cardiopulmonary exercise testing results	PHR	2	49	45	−0.9819 [−5.2474; 3.2836]	1.45	0.1470	Fixed	0.86	0.3549	0	0.9791	0.5067
	PSBP	2	49	45	7.5343 [0.4519; 14.6167]	−0.45	0.6519	Fixed	1.55	0.2130	35.5	–	–
	RHR	3	61	52	−2.8305 [−6.6556; 0.9947]	0.30	0.7628	Fixed	0.35	0.8386	0	–	–
	RSBP	2	31	26	1.2469 [−6.8507; 9.3446]	1.67	0.0946	Fixed	0.33	0.5636	0	–	–
Echocardiographic results	LVEF	14	456	442	6.6500 [5.4584; 7.8415]	10.94	<0.0001	Random	27.82	0.0096	53.3	0.3736	0.7152
	LVESV	6	142	132	−13.2522 [−18.7347; −7.7698]	−4.74	<0.0001	Random	13.49	0.0192	62.9	0.6239	0.5665
	LVEDV	6	142	132	−5.2938 [−13.8592; 3.2717]	−1.21	0.2258	Random	17.10	0.0043	70.8	1.4803	0.2129
	SWTSI	4	91	84	−0.3140 [−0.4740; −0.1540]	−3.85	0.0001	Random	12.68	0.0054	76.3	0.0149	0.9895
	LVESD	7	187	179	−0.5828 [−1.0852; −0.0804]	−2.27	0.0230	Random	420.88	<0.0001	98.6	0.0106	0.9920
	LVEDD	8	242	232	−0.4025 [−0.5483; −0.2566]	−5.41	<0.0001	Random	16.19	0.0235	56.8	2.5456	0.0438

*Random-effects model was used when the *P*-value for heterogeneity test <0.05, otherwise the fixed-effect model was used. ^†^*P*-value <0.05 is considered statistically significant for *Q* statistics. ^‡^Egger’s test to evaluate publication bias; *P*-value<0.05 is considered statistically significant.

### Echocardiographic results

**Figure 4:**
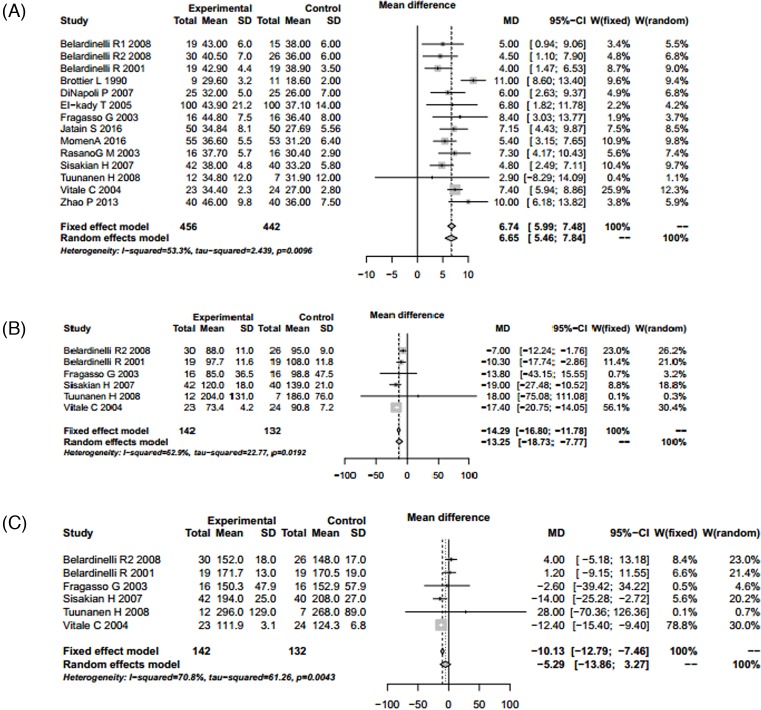
Forest plot for LVEF, LVESV, and LVEDV of TMZ group and control group (**A**) Forest plot for the improvement of LVEF. (**B**) Forest plot for LVESV reduction. (**C**) Forest plot for LVEDV reduction. Abbreviation: S.D., standard deviation.

**Figure 5:**
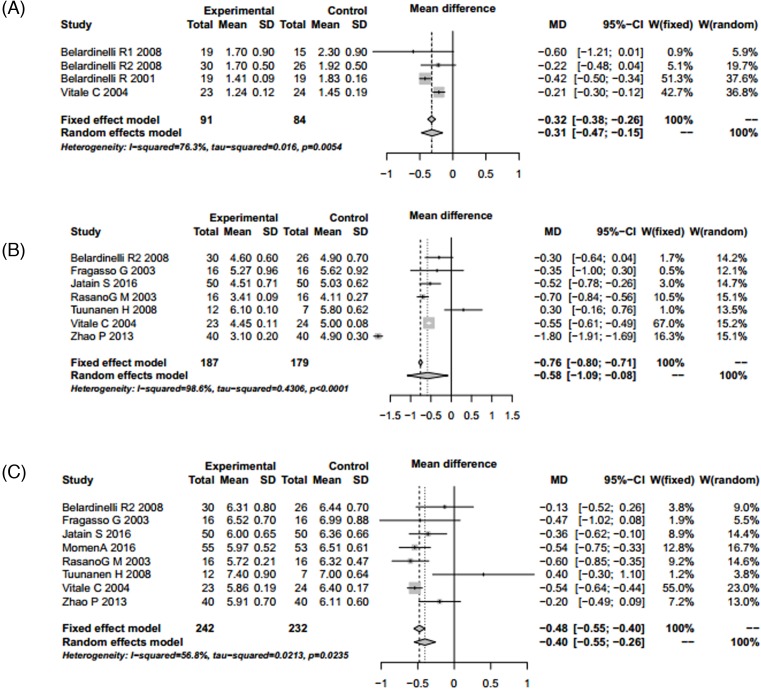
Forest plot for SWTSI, LVESD, and LVEDD of TMZ group and control group (**A**) Forest plot for the improvement of SWTSI. (**B**) Forest plot for LVESD reduction. (**C**) Forest plot for LVEDD reduction.

Indexes including LVEF, LVESV, LVEDV, SWTSI, LVESD, and LVEDD were conformed to the random utility model condition (*P*<0.05 and *I^2^* > 50%), and random utility model was used for merging. The results showed that LVEF (TMZ group 456, control group 442, MD = 6.65, 95% CI: 5.46 – 7.84, *Z* = 10.94, *P*<0.001), LVESV (TMZ group 142, control group 132, MD = −13.25, 95% CI: −18.73 to −7.77, *Z* = −4.74, *P*<0.001), LVEDV (TMZ group 142, control group 132, MD = −5.29, 95% CI: −13.86 to 3.27, *Z* = −1.21, *P*=0.2258), SWTSI (TMZ group 91, control group 84, MD = −0.31, 95% CI: −0.47 to −0.15, *Z* = −3.85, *P*=0.001), LVESD (TMZ group 187, control group 179, MD = −0.58, 95% CI: −1.09 to −0.08, *Z* = −2.27, *P*<0.001) and LVEDD (TMZ group 242, control group 232, MD = −0.40, 95% CI: −0.55 to −0.26, *Z* = −5.41, *P*=0.023). There were significant differences in LVEF, LVESV, SWTSI, LVESD, and LVEDD between the TMZ group and the control group. TMZ-treatment significantly increased level of LVEF, and reduced level of LVESV, SWTSI, LVESD, and LVEDD. Publication bias was assessed using the ‘Egger’s’ method. The results showed that there were no publication bias except for LVEDV (*t* = 2.5456, *P*=0.0438) (Table 3; Figures 4 and 5).

## Discussion

We performed a meta-analysis for TMZ effect on cardiomyopathy treatment, found that TMZ had effects on the treatment of ischemic cardiomyopathy and dilated cardiomyopathy, and improved clinical symptoms. TMZ initially used as an anti-ischaemic or ‘cytoprotective’ agent through regulating a metabolic pathway of switching cardiac metabolism from FFA to glucose oxidation, increasing glucose utilization, improving the rate of ATP production, optimizing energy, thereby reducing myocardial damage, and achieving purpose of myocardial protection [22]. FFA inhibitors could be used as metabolic modulators to protect the ischaemic myocardium, the effects of TMZ on cardiomyopathy were previously evaluated primarily for patients with ischemic heart failure [23]. TMZ could improve heart functions, which has been confirmed by clinical studies [24]. Through collecting these experimental results, our meta-analysis confirmed that TMZ significantly improved LVEF, LVESV, SWTSI, and LVESD, and decreased LVEDD level, which indicates that TMZ significantly improved left ventricular remodeling and systolic function in patients with cardiomyopathy.

At present, there are many researches on TMZ-treatment for cardiomyopathy. Some studies had shown that TMZ-treatment could improve left ventricular(LV) function and exercise tolerance, and reduce plasma levels of type-B natriuretic peptide, and cardiac troponin T levels in patients with ischemic cardiomyopathy [10, 25]. In addition, compared with TMZ or exercise training alone, TMZ combined ET could significantly improve functional capacity, LVEF, and endothelium-dependent dilation [9]. Moreover, TMZ-treatment could improve LV function, symptoms, glucose metabolism, endothelial function, and physical tolerance in patients with diabetic cardiomyopathy [26–28]. In idiopathic dilated cardiomyopathy patients, TMZ could increase cardiac function and have both cardiac and extracardiac metabolic effects [17, 29, 30]. TMZ could improve LV function and life quality in patients with coronary artery disease [20].

Improving cardiac function of patients with cardiomyopathy is the most important treatment target [31]. In the present study, the results showed that TMZ significantly improved left ventricular remodeling and systolic function. However, the difference in outcome caused by other factors could not be ignored. The results of heterogeneity test in the present study were significant, which might be induced by the following reasons: factors of different regions, such as living habits, environment, and the level of economic development; effects of gender, age, and other confounding factors.

In order to test the reliability of previous studies, the present study conducted a meta-analysis of TMZ for cardiomyopathy treatment to ensure the reliability of these conclusions. It has to be mentioned that there are some limitations in the present study. First, due to some incomplete data, no covariates were corrected, and no subgroup analysis was also performed. These potential confounding factors might affect the results of meta-analysis. Second, publication bias exists in LVEDD, which might have an impact on the synthetic results. Third, the included articles with four indicators of cardiopulmonary exercise testing results were too fewer; thereby, publication bias assessments could not be processed.

In conclusion, this meta-analysis showed that TMZ is effective for cardiomyopathy treatment, and a growing body of evidence supports that the potential role of TMZ in treating patients with heart failure. However, this conclusion still needs to be validated by larger-scale, higher-quality studies, or updated meta-analysis with more samples.

## Abbreviations

LVEDD: left ventricular end-diastolic diameter
LVEDV: left ventricular end-diastolic volume
LVEF: left ventricular ejection fraction
LVESD: left ventricular end-systolic diameter
LVESV: left ventricular end-systolic volume
MD: mean difference
PHR: peak heart rate
PSBP: peak systolic blood pressure
RHR: resting heart rate
RSBP: resting systolic blood pressure
SWTSI: systolic wall thickening score index
TMZ: trimetazidine

## References

[1] Pereira N.M.C., Barbosa M.M., Ribeiro A.L., Amorim Fenelon L.M. and Rocha M.O. (2010) Predictors of mortality in patients with dilated cardiomyopathy: relevance of chagas disease as an etiological factor. Revista Española De Cardiología 63, 788–797 10.1016/S1885-5857(10)70163-8 20609312

[2] Grundy M.P. (2002) The individual and the meeting. J. Emerg. Med. 29, 437–441

[3] de Haan T.R., Beersma M.F., Claas E.C., Oepkes D., Kroes A.C. and Walther F.J. (2007) Parvovirus B19 infection in pregnancy studied by maternal viral load and immune responses. Fetal Diagnosis Therapy 22, 55 10.1159/000095845 17003557

[4] Kantor P.F., Lucien A., Kozak R. and Lopaschuk G.D. (2000) The antianginal drug trimetazidine shifts cardiac energy metabolism from fatty acid oxidation to glucose oxidation by inhibiting mitochondrial long-chain 3-ketoacyl coenzyme A thiolase. Circ. Res. 86, 580 10.1161/01.RES.86.5.580 10720420

[5] Krebs M., Krssak M., Nowotny P., Weghuber D., Gruber S., Mlynarik V. (2001) Free fatty acids inhibit the glucose-stimulated increase of intramuscular glucose-6-phosphate concentration in humans. J. Clin. Endocrinol. Metab. 86, 2153 11344220 10.1210/jcem.86.5.7488

[6] Lau J.I., Ioannidis J.P. and Schmid C.H. (1997) Quantitative synthesis in systematic reviews. Ann. Intern. Med. 127, 820–826 10.7326/0003-4819-127-9-199711010-00008 9382404

[7] Feng R.N., Chen Z., Sun C.H. and Ying L. (2011) Meta-Analysis ofTNF308 G/A polymorphism and type 2 diabetes mellitus. PLoS ONE 6, e18480 10.1371/journal.pone.0018480 21494616 PMC3072982

[8] Belardinelli R., Cianci G., Gigli M., Mazzanti M. and Lacalaprice F. (2008) Effects of trimetazidine on myocardial perfusion and left ventricular systolic function in type 2 diabetic patients with ischemic cardiomyopathy. J. Cardiovasc. Pharmacol. 51, 611–615 10.1097/FJC.0b013e31817bdd66 18574390

[9] Belardinelli R., Lacalaprice F., Faccenda E. and Volpe L. (2008) Trimetazidine potentiates the effects of exercise training in patients with ischemic cardiomyopathy referred for cardiac rehabilitation. Eur. J. Cardiovasc. Prev. Rehabil. 15, 533 10.1097/HJR.0b013e328304feec 18797405

[10] Belardinelli R. and Purcaro A. (2001) Effects of trimetazidine on the contractile response of chronically dysfunctional myocardium to low-dose dobutamine in ischaemic cardiomyopathy. Eur. Heart J. 22, 2164 10.1053/euhj.2001.2653 11913478

[11] Brottier L., Barat J.L., Combe C., Boussens B., Bonnet J. and Bricaud H. (1990) Therapeutic value of a cardioprotective agent in patients with severe ischaemic cardiomyopathy. Eur. Heart J. 11, 207 10.1093/oxfordjournals.eurheartj.a059685 2318223

[12] Di N.P., Di G.P., Gaeta M.A., D’Apolito G. and Barsotti A. (2007) Beneficial effects of trimetazidine treatment on exercise tolerance and B-type natriuretic peptide and troponin T plasma levels in patients with stable ischemic cardiomyopathy. Am. Heart J. 154, 602.e1 10.1016/j.ahj.2007.06.033 17719313

[13] El-Kady T., El-Sabban K., Gabaly M., Sabry A. and Abdel-Hady S. (2005) Effects of trimetazidine on myocardial perfusion and the contractile response of chronically dysfunctional myocardium in ischemic cardiomyopathy. Am. J. Cardiovasc. Drugs 5, 271–278 10.2165/00129784-200505040-00006 15984909

[14] Fragasso G., Piatti P., Monti M.L., Palloshi A., Setola E., Puccetti P. (2003) Short- and long-term beneficial effects of trimetazidine in patients with diabetes and ischemic cardiomyopathy. Am. Heart J. 146, 854 10.1016/S0002-8703(03)00415-0 14597947

[15] Rosano Giuseppe M.C., Cristiana V., Barbara S., Giuseppe M. and Massimo F. (2003) Trimetazidine improves left ventricular function in diabetic patients with coronary artery disease: a double-blind placebo-controlled study. Cardiovasc. Diabetol. 2, 16 10.1186/1475-2840-2-16 14641923 PMC305354

[16] Kapoor A., Jatain S., Agarwal S.K., Pande S., Sinha A., Khanna R. (2014) Metabolic manipulation in dilated cardiomyopathy: assessing the role of trimetazidine. J. Cardiothoracic Surg. 66, A7 10.1016/j.ihj.2016.04.023 PMC5143816 27931551

[17] Momen A., Ali M., Karmakar P.K., Ali M.Z., Haque A., Khan M.R. (2016) Effects of sustained-release trimetazidine on chronically dysfunctional myocardium of ischemic dilated cardiomyopathy - Six months follow-up result. Indian Heart J. 68, 809 10.1016/j.ihj.2016.03.021 27931552 PMC5143824

[18] Sisakian H., Torgomyan A. and Barkhudaryan A. (2007) The effect of trimetazidine on left ventricular systolic function and physical tolerance in patients with ischaemic cardiomyopathy. Acta Cardiol. 62, 493 10.2143/AC.62.5.2023413 17982971

[19] Tuunanen H., Engblom E., Naum A., Någren K., Scheinin M., Hesse B. (2008) Trimetazidine, a metabolic modulator, has cardiac and extracardiac benefits in idiopathic dilated cardiomyopathy. Circulation 118, 1250 10.1161/CIRCULATIONAHA.108.778019 18765391

[20] Vitale C., Wajngaten M., Sposato B., Gebara O., Rossini P., Fini M. (2004) Trimetazidine improves left ventricular function and quality of life in elderly patients with coronary artery disease. Eur. Heart J. 25, 1814 10.1016/j.ehj.2004.06.034 15474696

[21] Zhao P., Zhang J., Yin X.G., Maharaj P., Narraindoo S., Cui L.Q. (2013) The effect of trimetazidine on cardiac function in diabetic patients with idiopathic dilated cardiomyopathy. Life Sci. 92, 633–638 10.1016/j.lfs.2012.03.015 22484413

[22] Hu B., Li W., Xu T., Chen T. and Guo J. (2011) Evaluation of trimetazidine in angina pectoris by echocardiography and radionuclide angiography: a meta-analysis of randomized, controlled trials. Clin. Cardiol. 34, 395 10.1002/clc.20888 21538382 PMC6652323

[23] Fragasso G., Spoladore R., Baratto F., Arioli F., Marinosci G. and Margonato A. (2008) New therapeutic strategies in heart failure: targeting free fatty oxidation. New Armenian Med. J. 2, 8–19

[24] Fragasso G., Rosano G., Sang H.B., Sisakian H., Napoli P.D., Alberti L. (2013) Effect of partial fatty acid oxidation inhibition with trimetazidine on mortality and morbidity in heart failure: results from an international multicentre retrospective cohort study. Int. J. Cardiol. 163, 320–325 10.1016/j.ijcard.2012.09.123 23073279

[25] Di N.P., Di G.P., Gaeta M.A., D’Apolito G. and Barsotti A. (2007) Beneficial effects of trimetazidine treatment on exercise tolerance and B-type natriuretic peptide and troponin T plasma levels in patients with stable ischemic cardiomyopathy. Am. Heart J. 154, 1–5 10.1016/j.ahj.2007.06.033 17719313

[26] Wang X.Y. and Zhao J.L. (2013) Study on the effect of trimetazidine on myocardial perfusion and left ventricular systolic function in type 2 diabetic patients with ischemic cardiomyopathy. Med. J. West China. 25, 1078–1080 10.1097/FJC.0b013e31817bdd66 18574390

[27] Rosano G.M., Vitale C., Sposato B., Mercuro G. and Fini M. (2003) Trimetazidine improves left ventricular function in diabetic patients with coronary artery disease: a double-blind placebo-controlled study. Cardiovasc. Diabetol. 2, 1–9 10.1186/1475-2840-2-16 14641923 PMC305354

[28] Zhao P., Zhang J., Yin X.G., Maharaj P., Narraindoo S., Cui L.Q. (2013) The effect of trimetazidine on cardiac function in diabetic patients with idiopathic dilated cardiomyopathy. Life Sci. 92, 633–638 10.1016/j.lfs.2012.03.015 22484413

[29] Kapoor A., Jatain S., Agarwal S.K., Pande S., Sinha A., Khanna R. (2015) Metabolic manipulation in dilated cardiomyopathy: assessing the role of trimetazidine. Indian Heart J. 10, A7 10.1016/j.ihj.2016.04.023 PMC5143816 27931551

[30] Tuunanen H., Engblom E., Naum A., Någren K., Scheinin M., Hesse B. (2008) Trimetazidine, a metabolic modulator, has cardiac and extracardiac benefits in idiopathic dilated cardiomyopathy. Circulation 118, 1250 10.1161/CIRCULATIONAHA.108.778019 18765391

[31] Vizzardi E., Pina P.D., Caretta G., Bonadei I., Sciatti E., Lombardi C. (2012) The effect of aldosterone-antagonist therapy on aortic elastic properties in patients with nonischemic dilated cardiomyopathy. J. Cardiovasc. Med. 16, 597–602 10.2459/JCM.0000000000000102 24978872

